# A Practical Treatise on the Diseases of Children

**Published:** 1846-05

**Authors:** 


ART. IX.—*A Practical Treatise on the Diseases of Children.* By JAS. STEWART, M.D., A.M.; Fellow of the College of Physicians and Surgeons; one of the Consulting Physicians of the Northern Dispensary of the City of New York; Late Physician to the Orphan Asylum, &c., &c. Third edition, carefully revised and enlarged. New York: Harper & Brothers, 82 Cliffe street. 1845. 8vo. pp. 544.

When a work has passed to the third edition, it may be supposed to have reached a point at which its author may be careless of criticism. The public has then set the broad seal of its approbation upon it, and it has passed beyond the reach of the press. This treatise of Dr. Stewart's is in that enviable position, and, as we should judge, without much of that laudation by which many a work has been brought into temporary favor. In this edition the Harpers have given us a fine specimen of their mechanical skill; they have issued the work in a very substantial and tasteful style, as if conscious that they were sending abroad a book that would be used. The paper and binding will bear to be handled, while its matter will instruct its readers in one of the most interesting branches of the healing art. The diseases of children—so rife, often so sudden in their incursion, and so rapid in their progress—with what force do they appeal to our sympathies! We rejoice at the appearance of every new work devoted to their special elucidation. We cannot be too sedulous in the study of their nature and cure.

This elaborate work is for sale at the bookstores of Mr. Maxwell and Mr. Prescott.

